# Self-Normalized Cramér Type Moderate Deviations for Pooled Estimation in Branching Processes in a Random Environment

**DOI:** 10.3390/e28091018

**Published:** 2026-09-11

**Authors:** Quanzhen Yao, Mengyu Li

**Affiliations:** School of Mathematics and Statistics, Northeastern University at Qinhuangdao, Qinhuangdao 066004, China; 202412467@stu.neuq.edu.cn

**Keywords:** branching process in random environment, pooled estimator, self-normalized Cramér deviation, martingale differences, efficiency gain, confidence interval, 60G42, 60F10, 60J80

## Abstract

We study the estimation of the offspring mean of a supercritical branching process in a random environment when multiple conditionally independent populations evolve in a common environment. Extending the single-population self-normalized Cramér moderate deviation theory to this multi-population setting, we introduce a pooled Lotka–Nagaev estimator and construct its associated martingale difference sequence. The core of the analysis is an exact decomposition of the pooled conditional variance, which, owing to the shared environment and conditional independence of the populations, separates the environmental and demographic sources of variability. This decomposition reveals a structural dichotomy: the environmental variance is undiluted by pooling, while the demographic variance is attenuated at a rate proportional to the inverse square of the number of populations. Verifying the two conditions of the martingale moderate-deviation theorem yields self-normalized Cramér moderate deviations for the pooled Student t-statistic, together with a Berry–Esseen bound, a moderate deviation principle, and confidence intervals for the offspring mean. The resulting pooling efficiency gain, in which the demographic variance decays inversely with the number of populations while the environmental variance forms an irreducible floor, has no analogue in any single-population framework and is confirmed by Monte Carlo simulation.

## 1. Introduction

### 1.1. Background

A branching process in a random environment (BPRE) models the growth of a population whose reproduction law fluctuates randomly from one generation to the next. Formally, let $\xi =(\xi_{0},\xi_{1},\ldots )$ be a sequence of i.i.d. random variables, the *environment*; given ξ, the population sizes evolve as

(1) $$Z_{0}=1,\;Z_{n+1}=\sum_{i=1}^{Z_{n}}X_{n,i},$$

where the offspring numbers $X_{n,i}$ are conditionally independent with a common law $\left\{p_{k}\left(\xi_{n}\right)\right\}$ determined by the environment at generation *n*. Introduced by Smith and Wilkinson [1] and further developed by Athreya and Karlin [2], the BPRE has become a canonical model for populations subject to environmental stochasticity, and its limit theory is now extensive.

A central problem in the statistical inference of BPRE is the estimation of the *offspring mean* $m=E[m\left(\xi_{0}\right)]$, where $m\left(\xi_{n}\right)=\sum_{r\geq 0}r\;p_{r}\left(\xi_{n}\right)$ is the conditional mean number of offspring per individual. The classical estimator is the Lotka–Nagaev estimator [3,4]

(2) $${\hat{m}}_{n_{0},n}=\frac{1}{n}\sum_{k=n_{0}}^{n_{0}+n-1}\frac{Z_{k+1}}{Z_{k}},$$

which averages the successive one-step growth ratios. Its asymptotic normality follows from the martingale central limit theorem of Scott [5]; moreover, the exact variance decomposition

(3) $$\mathrm{Var}\;\left(\frac{Z_{k+1}}{Z_{k}}\;|\;F_{k}\right)=\sigma^{2}+\frac{\tau^{2}}{Z_{k}},$$

where $F_{k}=\sigma \left(Z_{0},\ldots ,Z_{k}\right)$ denotes the natural filtration of the process, separating the environmental variance $\sigma^{2}=\mathrm{Var}\left(m\left(\xi_{0}\right)\right)$ from the demographic variance $\tau^{2}=E\left[\mathrm{Var}\left(X_{0,1}|\xi_{0}\right)\right]$, goes back to Dion and Esty [6]. This single-population identity is precisely the case L=1 of the conditional variance formula we establish below; we do not claim it as new.

Beyond first-order asymptotics, the fine fluctuation behavior of a single BPRE has been studied in depth: harmonic moments and large deviations of the Lotka–Nagaev estimator [7,8], upper and lower large deviations of the population size [9,10], and Berry–Esseen bounds and Cramér-type expansions [11,12]. All these works, however, concern a single BPRE; none addresses the multi-population pooled setting.

On the methodological side, Cramér-type moderate deviations quantify the relative error of the normal approximation in the tails. Since Cramér [13], the theory has developed from independent sums to the powerful *self-normalized* framework [14,15,16], which dispenses with knowledge of the variance and requires only finite moments, and further to martingales [17,18,19]. In particular, Fan and Shao [19] (Section 3.3) established self-normalized Cramér moderate deviations for the Lotka–Nagaev *t*-statistic of a *single* BPRE, providing the theoretical backbone on which the present work builds.

When *L* independent populations share the same environment, the single-population theory no longer applies directly. The reason is twofold: first, the populations are coupled through the common environment, producing non-trivial cross-dependence terms absent from the single-process case; second, naively applying the single-population result to each trajectory ignores the efficiency gain achievable by pooling. The present paper addresses exactly these two points: we derive the conditional variance formula that captures the cross-process coupling, we verify the relevant martingale conditions in the pooled setting, and we quantify the resulting improvement in estimation accuracy. This leads naturally to the multi-population framework described in the next section. In what follows, we formalize this setting by introducing *L* conditionally independent BPREs that share a common environment (Section 2), construct the pooled Lotka–Nagaev estimator, and derive the conditional variance formula that captures the cross-process coupling (Section 3). The resulting moderate-deviation theorem then quantifies precisely how pooling improves estimation accuracy relative to the single-population benchmark, and the numerical study in Section 4 confirms the predicted efficiency gain.

### 1.2. Motivation and Relation to Concurrent Work

In many applications, one observes not a single population but several independent populations evolving in the *same* environment: sub-populations monitored in one forest, replicate cultures in one laboratory, or parallel experimental arms subject to common external conditions. When these populations share the same offspring mean *m*, pooling their data should estimate *m* more accurately than any single trajectory. This paper aims to make this intuition precise and quantify the resulting efficiency gain.

It is important to locate our contribution among the existing extensions of the self-normalized Cramér framework for BPRE, which proceed along two distinct directions. Fan and Shao [19] (Section 3.3) treat the single-population baseline (L=1, no immigration). Wang and Xiao [20] extend the framework to a single Galton–Watson process *with immigration* in a random environment, where the new structural quantity is the immigration variance ${\bar{v}}^{2}$; see also Wang and Peng [21] for the constant-environment case. The present paper pursues a third, orthogonal direction: *L* conditionally independent populations *without immigration*, pooled together. Immigration enriches the within-population dynamics, whereas pooling introduces a cross-sectional dimension *L*; the efficiency-gain phenomenon we analyze has no analogue in any single-population setting, with or without immigration.

We note that an earlier line of work on branching processes with a random or increasing number of ancestors [22] also involves multiple lineages, but in a fixed environment rather than a shared random one, and does not address self-normalized moderate deviations. Our shared-random-environment structure is what couples the populations and produces the constant cross-terms central to our analysis.

### 1.3. Our Contribution

The contributions of this paper are the following.

1. We introduce the **pooled Lotka–Nagaev estimator** for *L* conditionally independent BPREs sharing a common environment,
  (4) $${\hat{m}}_{n_{0},n}^{\left(L\right)}=\frac{1}{Ln}\sum_{j=1}^{L}\sum_{k=n_{0}}^{n_{0}+n-1}\frac{Z_{k+1}^{\left(j\right)}}{Z_{k}^{\left(j\right)}},$$
  and its associated martingale difference sequence.
2. We derive the **pooled conditional variance formula** (Theorem 1),
  (5) $$E\left[{\left({\tilde{\eta }}_{k+1}^{\left(L\right)}\right)}^{2}|F_{k}\right]=\sigma^{2}+\frac{\tau^{2}}{L^{2}}\sum_{j=1}^{L}\frac{1}{Z_{k}^{\left(j\right)}},$$
  which generalizes the single-population identity of Dion and Esty [6]. The genuinely new mathematical difficulty addressed here is the cross-dependence induced by the shared environment: had the *L* populations been independent, the environmental variance would be diluted by the factor 1/L under pooling. Because they share the same environment, however, all L(L−1) cross-terms equal $\sigma^{2}$ so that the environmental variance is not diluted at all, and only the demographic term $\tau^{2}$ is attenuated at rate $1/L^{2}$. This sharp dichotomy between the two variance components is the central finding of the paper.
3. We establish **self-normalized Cramér moderate deviations** for the pooled Student’s *t*-statistic (Theorem 2), valid over the same range $0\leq x=o\left(n^{\rho /(4+2\rho )}\right)$ as in the single-population case, together with a uniform Berry–Esseen bound, a moderate deviation principle, and confidence intervals for *m*.
4. We **quantify the pooling efficiency gain**: the demographic component of the variance decays like 1/L while the environmental component $\sigma^{2}/n$ is irreducible. This dichotomy, absent in every single-population framework, is confirmed by simulation in Section 4.

Our proof strategy is deliberately economical: rather than re-deriving the moderate-deviation machinery, we show that the pooled martingale differences satisfy the two conditions of Fan and Shao [19] (Theorem 2.1) with exactly the same parameter orders as in the single-population case, so that their theorem applies directly. The mathematical novelty therefore resides in the conditional variance computation and the verification of these conditions in the multi-population setting, and in the resulting efficiency analysis.

### 1.4. Structure of the Paper

Section 2 presents the model and notation. Section 3 contains the main results: the pooled estimator, the conditional variance formula, the verification of the two conditions, and the moderate-deviation theorem with its corollaries. Section 4 reports a Monte Carlo study confirming the theory and, in particular, the 1/L efficiency gain. Section 5 contains the proofs. Section 6 discusses extensions and open problems.

## 2. Model and Notation

### 2.1. Branching Processes in a Random Environment

Let $\xi =(\xi_{0},\xi_{1},\ldots )$ be a sequence of independent and identically distributed (i.i.d.) random variables taking values in some measurable space Θ; we call ξ the *random environment*. Each realization of $\xi_{n}$ determines a probability distribution $\left\{p_{k}\left(\xi_{n}\right):k\in N\right\}$ on N={0,1,2,…}, with probability generating function

(6) $$f_{\xi_{n}}\left(s\right)=\sum_{k\geq 0}p_{k}\left(\xi_{n}\right)\;s^{k},\;s\in \left[0,1\right].$$

Given the environment ξ, a branching process ${\left(Z_{n}\right)}_{n\geq 0}$ evolves according to (1). We denote by $P_{\xi }$ the *quenched law* (the conditional probability given the environment ξ), by Υ the law of ξ, and by $P\left(dx,d\xi \right)=P_{\xi }\left(dx\right)\;\Upsilon \left(d\xi \right)$ the *annealed law*. The corresponding expectations are written $E_{\xi }$ and E, respectively.

The conditional and unconditional offspring means are

(7) $$m\left(\xi_{n}\right)=E_{\xi }X_{n,i}=\sum_{k\geq 0}k\;p_{k}\left(\xi_{n}\right),\;m=E\left[m\left(\xi_{0}\right)\right].$$

The offspring mean *m* is the parameter to be estimated. Following Fan and Shao [19], we introduce the variance parameters

(8) $$\begin{array}{cccc} \sigma^{2} & =E\left[{\left(m\left(\xi_{0}\right)-m\right)}^{2}\right] & \; & (\mathrm{environmental}\;\mathrm{variance}), \end{array}$$

(9) $$\begin{array}{cccc} \tau^{2} & =E\left[{\left(Z_{1}-m\left(\xi_{0}\right)\right)}^{2}\right] & \; & (\mathrm{demographic}\;\mathrm{variance}), \end{array}$$

(10) $$\begin{array}{cccc} \upsilon^{2} & =E\left[{\left(Z_{1}-m\right)}^{2}\right] & \; & (\mathrm{total}\;\mathrm{variance}\;\mathrm{of}\;Z_{1}), \end{array}$$

together with the criticality parameter $\mu =E[\ln m\left(\xi_{0}\right)]$. Since $Z_{1}-m=\left(Z_{1}-m\left(\xi_{0}\right)\right)+\left(m\left(\xi_{0}\right)-m\right)$ and the cross-expectation vanishes, one has the *variance decomposition*

(11) $$\upsilon^{2}=\tau^{2}+\sigma^{2},$$

which separates the demographic (within-environment) and environmental sources of variability; see Dion and Esty [6]. Throughout, we assume 0<υ,σ<∞ and μ>0, so that the process is supercritical.

Finally, let

(12) $$V_{n}=\frac{Z_{n}}{\prod_{i=0}^{n-1}m\left(\xi_{i}\right)},\;n\geq 1,\;V_{0}=1.$$

Under mild moment conditions, $\left(V_{n}\right)$ is a nonnegative martingale converging almost surely and in $L^{1}$ to a limit *V* with P(V>0)=1 (see Athreya and Karlin [2]).

### 2.2. The Multi-Population Model

We now introduce the object of study: *L* branching processes evolving in a *common* random environment. Here, L≥1 is a fixed integer denoting the number of populations; it plays a role entirely distinct from the offspring mean *m*, and the two should not be confused.

**Notation.** Throughout the paper, *m* always denotes the offspring mean, following the convention of the branching-process literature, whereas *L* denotes the number of pooled populations. Superscripts (j), 1≤j≤L, index the individual populations.

In the same environment ξ, let ${\left(Z_{n}^{\left(1\right)}\right)}_{n\geq 0},\ldots ,{\left(Z_{n}^{\left(L\right)}\right)}_{n\geq 0}$ be defined by

(13) $$Z_{0}^{\left(j\right)}=1,\;Z_{n+1}^{\left(j\right)}=\sum_{i=1}^{Z_{n}^{\left(j\right)}}X_{n,i}^{\left(j\right)},\;j=1,\ldots ,L,$$

where, conditionally on ξ, the entire family

$$\left\{X_{n,i}^{\left(j\right)}:n\geq 0,\;i\geq 1,\;1\leq j\leq L\right\}$$

is independent, and each $X_{n,i}^{\left(j\right)}$ has distribution $\left\{p_{k}\left(\xi_{n}\right):k\in N\right\}$.

Thus, the *L* populations share the same environmental sequence ξ but reproduce independently once the environment is given. In particular, for each *n*, the variables $Z_{n+1}^{\left(1\right)},\ldots ,Z_{n+1}^{\left(L\right)}$ are conditionally independent given $\left(\xi_{n},Z_{n}^{\left(1\right)},\ldots ,Z_{n}^{\left(L\right)}\right)$, and each has the same conditional offspring mean $m(\xi_{n})$. This *shared-environment/conditional-independence* structure is what produces the constant cross-terms in the conditional variance formula of Section 3 and is the mechanism behind the pooling efficiency gain.

### 2.3. Assumptions

We impose the following conditions, adapted from [12,19].

(H1) $p_{0}\left(\xi_{0}\right)=0$ almost surely, so that each individual has at least one offspring and the process survives (supercritical regime).
(H2) $E\;\left[\frac{Z_{1}}{m(\xi_{0})}\ln^{+}Z_{1}\right]<\infty$, where $\ln^{+}x=\ln\left(x\vee 1\right)$; this ensures $V_{n}\to V$ in $L^{1}$ with V>0 a.s.
(H3) There exist constants p>1 and $\eta_{0}\in \left(0,1\right)$ such that $E\left[{\left(\theta_{0}\left(p\right)\right)}^{\eta_{0}}\right]<\infty$, where $\theta_{0}\left(p\right)=Z_{1}^{\;p}/{\left(m(\xi_{0})\right)}^{p}$. This harmonic-moment condition guarantees $E[V^{-\alpha }]<\infty$ for some α>0 (cf. [12]).
(H4) There exists ρ∈(0,1] such that $E|Z_{1}-m\left(\xi_{0}\right){|}^{2+\rho }+E{\left|m\left(\xi_{0}\right)-m\right|}^{2+\rho }<\infty$ (moment condition for moderate deviations).
(H5) L∈N is fixed and does not grow with *n*.

Assumptions (H1)–(H4) concern a single population and are exactly those used in [19] (Section 3.3); since the *L* populations are identically distributed, they hold simultaneously for every *j*. Assumption (H5) fixes the scope of the present paper; the regime $L=L_{n}\to \infty$ is discussed in Section 6.

We comment on the strength of (H1). The condition $p_{0}\left(\xi_{0}\right)=0$ a.s. means that every individual produces at least one offspring, so the population size is non-decreasing and never vanishes. Consequently the ratios $Z_{k+1}/Z_{k}$ and the inverse quantities $1/Z_{k}$ are always well defined, and the harmonic-moment and tail estimates of Lemmas A1 and A2 (see the Appendix A) take their simplest form. This assumption, inherited from Fan and Shao [19], is strictly stronger than the usual supercritical regime, in which $p_{0}>0$ is permitted and extinction occurs with positive probability. Our results therefore concern the no-extinction idealization, while extending them to the case $p_{0}>0$ would require conditioning on non-extinction, a standard but more delicate route in the BPRE literature, which we leave to future work.

### 2.4. Filtration and Conventions

For the pooled analysis, we work with the joint filtration generated by the environment and all *L* populations:

(14) $$F_{n_{0}}=\left\{\emptyset ,\Omega \right\},\;F_{k+1}=\sigma \left\{\xi_{i-1},\;Z_{i}^{\left(1\right)},\ldots ,Z_{i}^{\left(L\right)}:n_{0}\leq i\leq k+1\right\},\;k\geq n_{0}.$$

Here, $n_{0}\geq 0$ is a fixed starting generation; taking $n_{0}>0$ accommodates the practical situation in which the early data ${\left(Z_{i}^{\left(j\right)}\right)}_{0\leq i<n_{0}}$ are missing (cf. [19], Remark 3.1).

We use *c* and $c_{\rho }$ to denote generic positive constants, the latter depending only on ρ (and on the model parameters), whose values may change from line to line. We write Φ for the standard normal distribution function.

## 3. Main Result

### 3.1. Pooled Estimator and Martingale Construction

Recall that for a single population, the offspring mean *m* is estimated by the Lotka–Nagaev estimator [3,4]. Pooling the *L* populations, we define the *pooled Lotka–Nagaev estimator*

(15) $${\hat{m}}_{n_{0},n}^{\left(L\right)}=\frac{1}{Ln}\sum_{j=1}^{L}\sum_{k=n_{0}}^{n_{0}+n-1}\frac{Z_{k+1}^{\left(j\right)}}{Z_{k}^{\left(j\right)}},$$

which averages the one-step growth ratios over both time (*n* generations) and the *L* populations. Taking L=1 recovers the estimator of [19] (Section 3.3).

To study ${\hat{m}}_{n_{0},n}^{\left(L\right)}$ we introduce the martingale difference sequence

(16) $${\tilde{\eta }}_{k+1}^{\left(L\right)}=\frac{1}{L}\sum_{j=1}^{L}\frac{Z_{k+1}^{\left(j\right)}}{Z_{k}^{\left(j\right)}}-m,\;k=n_{0},\ldots ,n_{0}+n-1,$$

so that ${\hat{m}}_{n_{0},n}^{\left(L\right)}-m=\frac{1}{n}\sum_{k=n_{0}}^{n_{0}+n-1}{\tilde{\eta }}_{k+1}^{\left(L\right)}$.

**Proposition 1.**  *Under (H1)–(H5), ${\left({\tilde{\eta }}_{k+1}^{\left(L\right)},F_{k+1}\right)}_{k\geq n_{0}}$ is a sequence of martingale differences.*

**Proof.**  Measurability follows directly from (14). For each *j*, since $Z_{k+1}^{\left(j\right)}=\sum_{i=1}^{Z_{k}^{\left(j\right)}}X_{k,i}^{\left(j\right)}$ with $X_{k,i}^{\left(j\right)}$ conditionally independent of $F_{k}$ given $\xi_{k}$, and $\xi_{k}$ independent of $F_{k}$,

$$E\;\left[\frac{Z_{k+1}^{\left(j\right)}}{Z_{k}^{\left(j\right)}}\;|\;F_{k}\right]=\frac{1}{Z_{k}^{\left(j\right)}}\;E\;\left[\sum_{i=1}^{Z_{k}^{\left(j\right)}}X_{k,i}^{\left(j\right)}\;|\;F_{k}\right]=\frac{1}{Z_{k}^{\left(j\right)}}\cdot Z_{k}^{\left(j\right)}\;E\left[m\left(\xi_{k}\right)\right]=m.$$

Averaging over j=1,…,L and subtracting *m* gives $E[{\tilde{\eta }}_{k+1}^{\left(L\right)}|F_{k}]=0$. □

### 3.2. Conditional Variance Formula

The following identity is the central computation of the paper. For 1≤j≤L, write

(17) $$A_{k}^{\left(j\right)}=\frac{Z_{k+1}^{\left(j\right)}}{Z_{k}^{\left(j\right)}}-m,\;\mathrm{so}\;\mathrm{that}\;{\tilde{\eta }}_{k+1}^{\left(L\right)}=\frac{1}{L}\sum_{j=1}^{L}A_{k}^{\left(j\right)}.$$

**Theorem 1.**  *Under (H1)–(H5), for every $k\geq n_{0}$,*

(18) $$E\left[{\left({\tilde{\eta }}_{k+1}^{\left(L\right)}\right)}^{2}|F_{k}\right]=\sigma^{2}+\frac{\tau^{2}}{L^{2}}\sum_{j=1}^{L}\frac{1}{Z_{k}^{\left(j\right)}}.$$

The proof is given in Section 5.1; the mechanism is as follows. Expanding the square produces *L* diagonal terms and L(L−1) cross-terms:

(19) $$E\left[{\left({\tilde{\eta }}_{k+1}^{\left(L\right)}\right)}^{2}|F_{k}\right]=\frac{1}{L^{2}}\sum_{j=1}^{L}E\left[{\left(A_{k}^{\left(j\right)}\right)}^{2}|F_{k}\right]+\frac{1}{L^{2}}\sum_{i\neq j}E\left[A_{k}^{\left(i\right)}A_{k}^{\left(j\right)}|F_{k}\right].$$

Each diagonal term equals $\tau^{2}/Z_{k}^{\left(j\right)}+\sigma^{2}$ (the single-population identity of [6]). Each cross-term, by the shared-environment and conditional-independence structure, equals the constant $\sigma^{2}$:

$$E\left[A_{k}^{\left(i\right)}A_{k}^{\left(j\right)}|F_{k}\right]=E\;\left[E\left[A_{k}^{\left(i\right)}|\xi_{k},F_{k}\right]\;E\left[A_{k}^{\left(j\right)}|\xi_{k},F_{k}\right]\;|\;F_{k}\right]=E\left[{\left(m\left(\xi_{k}\right)-m\right)}^{2}\right]=\sigma^{2}.$$

Collecting the *L* diagonal and L(L−1) cross contributions, the $\sigma^{2}$ pieces combine to $L^{2}\sigma^{2}$, which, after division by $L^{2}$, yields (18).

Formula (18) exhibits two structural features that drive the results below.

1. **The environmental variance is undiluted.** The coefficient of $\sigma^{2}$ is exactly 1, independent of *L*: pooling cannot reduce the irreducible uncertainty coming from the shared environment.
2. **The demographic variance is attenuated at rate $1/L^{2}$.** The fluctuation term $\tau^{2}/L^{2}\sum_{j}1/Z_{k}^{\left(j\right)}$ decreases as *L* grows; in the symmetric case $Z_{k}^{\left(1\right)}\approx \cdots \approx Z_{k}^{\left(L\right)}$, it is of order $\tau^{2}/\left(LZ_{k}\right)$, a factor *L* smaller than in the single-population case. Setting L=1 recovers $\sigma^{2}+\tau^{2}/Z_{k}$ [19]. As *L* increases, the fluctuation term diminishes. The limit L→∞, understood only as a formal benchmark lying outside the fixed-*L* framework imposed by (H5), would send this term to 0, leaving only the constant $\sigma^{2}$.

### 3.3. Verification of Conditions (A1) and (A2)

Our route to moderate deviations is Theorem 2.1 of [19], which applies to a self-normalized martingale $W_{n}=S_{n}/\sqrt{{\left[S\right]}_{n}}$, provided the martingale differences satisfy two conditions, denoted (A1) and (A2). In the present notation, with $B_{n}^{2}=n\sigma^{2}$, these read as follows:

(A1) there exists $\delta_{n}\in \left(0,1/4\right]$ such that for all x>0, $\;P\left({|\langle S\rangle }_{n}-B_{n}^{2}|\geq xB_{n}^{2}\right)\leq c\exp\left(-x\delta_{n}^{-2}\right)$;
(A2) there exist ρ∈(0,1] and $\gamma_{n}\in \left(0,1/4\right]$ such that $\;E[|{\tilde{\eta }}_{k+1}^{\left(L\right)}{|}^{2+\rho }|F_{k}]\leq {\left(\gamma_{n}B_{n}\right)}^{\rho }\;E\left[{\left({\tilde{\eta }}_{k+1}^{\left(L\right)}\right)}^{2}|F_{k}\right]$.

The next two lemmas verify these for the pooled martingale; their proofs are in Section 5.

**Lemma 1**  (Condition (A2))**.** *Under (H1)–(H5), there is a constant $c_{1}>0$ such that for all k,*

$$E\left[|{\tilde{\eta }}_{k+1}^{\left(L\right)}{|}^{2+\rho }|F_{k}\right]\leq c_{1}\;E\left[{\left({\tilde{\eta }}_{k+1}^{\left(L\right)}\right)}^{2}|F_{k}\right].$$

*Consequently (A2) holds with $\gamma_{n}≍n^{-1/2}$, of the same order as in the single-population case.*

**Lemma 2**  (Condition (A1))**.** *Under (H1)–(H5), condition (A1) holds with $\delta_{n}^{2}≍\frac{\ln n}{n}$. In fact, $\delta_{n}^{2}$ is proportional to 1/L, so the concentration is sharpened as L grows, while the order $\delta_{n}^{2}≍\ln n/n$ remains unchanged.*

### 3.4. Pooled Student’s t-Statistic

The self-normalized statistic of primary interest is the pooled Student’s *t*-statistic

(20) $$N_{n_{0},n}^{\left(L\right)}=\frac{\sqrt{n}\;\left({\hat{m}}_{n_{0},n}^{\left(L\right)}-m\right)}{\sqrt{\frac{1}{n-1}\sum_{k=n_{0}}^{n_{0}+n-1}\;{\left(\frac{1}{L}\sum_{j=1}^{L}\frac{Z_{k+1}^{\left(j\right)}}{Z_{k}^{\left(j\right)}}-{\hat{m}}_{n_{0},n}^{\left(L\right)}\right)}^{\;2}}},$$

which is the pooled analogue of the *t*-statistic in [19]. Since the sample variance in the denominator is built from the same martingale differences ${\tilde{\eta }}_{k+1}^{\left(L\right)}$, the classical relation of Chung [23] expresses $N_{n_{0},n}^{\left(L\right)}$ in terms of the self-normalized sum $W_{n}^{\left(L\right)}=\left(\sum_{k}{\tilde{\eta }}_{k+1}^{\left(L\right)}\right)/{\left(\sum_{k}{\left({\tilde{\eta }}_{k+1}^{\left(L\right)}\right)}^{2}\right)}^{1/2}$; this reduction is carried out in Section 5.4.

### 3.5. Main Theorem

**Theorem 2.**  *Assume (H1)–(H5). Then, for all $n_{0}\geq 0$ and $0\leq x=o(\sqrt{n/\ln n})$,*

(21) $$\left|\ln\frac{P(N_{n_{0},n}^{\left(L\right)}\geq x)}{1-\Phi (x)}\right|=O\;\left(\frac{x^{2+\rho }}{n^{\rho /2}}+\frac{1+x}{n^{\rho (2-\rho )/8}\;\left(1+x^{\rho (2+\rho )/4}\right)}\right),$$

*where the implied constant depends only on ρ and the model parameters, not on n or x. For the relative error to tend to 0, x must be restricted to the narrower range $o(n^{\rho /(4+2\rho )})$; under this restriction, the leading term $x^{2+\rho }/n^{\rho /2}$ in the bound *(21)* is o(1), yielding the following.*

*In particular,*

(22) $$\frac{P(N_{n_{0},n}^{\left(L\right)}\geq x)}{1-\Phi (x)}=1+o\left(1\right)$$

*uniformly for $0\leq x=o\left(n^{\rho /(4+2\rho )}\right)$ as n→∞. The same statements hold with $P(N_{n_{0},n}^{\left(L\right)}\geq x)$ and 1−Φ(x) replaced by $P(N_{n_{0},n}^{\left(L\right)}\leq -x)$ and Φ(−x), respectively.*

### 3.6. Corollaries and Remarks

**Corollary 1**  (Uniform Berry–Esseen bound)**.** *Under (H1)–(H5),*

$$\underset{x\in R}{\sup}|P\left(N_{n_{0},n}^{\left(L\right)}\leq x\right)-\Phi \left(x\right)|=O\;\left(\frac{\ln n}{\sqrt{n}}\right).$$

**Corollary 2**  (Moderate deviation principle)**.** *Let ${\left(a_{n}\right)}_{n\geq 1}$ satisfy $a_{n}\to \infty$ and $a_{n}\sqrt{\ln n/n}\to 0$. Then, for every Borel set B⊆R,*

$$-\underset{x\in B^{\circ }}{\inf}\frac{x^{2}}{2}\leq \underset{n\to \infty }{lim inf}\frac{1}{a_{n}^{2}}\ln P\;\left(\frac{N_{n_{0},n}^{\left(L\right)}}{a_{n}}\in B\right)\leq \underset{n\to \infty }{lim sup}\frac{1}{a_{n}^{2}}\ln P\;\left(\frac{N_{n_{0},n}^{\left(L\right)}}{a_{n}}\in B\right)\leq -\underset{x\in \bar{B}}{\inf}\frac{x^{2}}{2}.$$

**Corollary 3**  (Confidence interval for *m*)**.** *Under (H1)–(H5), an asymptotic level-(1−α) confidence interval for the offspring mean m is*

$${\hat{m}}_{n_{0},n}^{\left(L\right)}\pm \frac{z_{\alpha /2}}{\sqrt{n}}\;{\left(\frac{1}{n-1}\sum_{k=n_{0}}^{n_{0}+n-1}\;{\left(\frac{1}{L}\sum_{j}Z_{k+1}^{\left(j\right)}/Z_{k}^{\left(j\right)}-{\hat{m}}_{n_{0},n}^{\left(L\right)}\right)}^{2}\right)}^{1/2},$$

*where $z_{\alpha /2}$ is the upper α/2 quantile of N(0,1); by *(22)*, its coverage error tends to 0 faster than any power of n within the moderate-deviation range.*

**Remark 1.**  *Setting L=1 recovers [19] (Theorem 3.3). The moderate-deviation range $o\left(n^{\rho /(4+2\rho )}\right)$ is of the same order as in the single-population case; the effect of L>1 is on the constants, through the $1/L^{2}$ attenuation of the demographic term in *(18)*, which sharpens the concentration of the conditional variance and thereby improves the finite-sample normal approximation. This efficiency gain has no counterpart in any single-population framework, with or without immigration (cf. [20]).*

## 4. Numerical Studies

We report a Monte Carlo study that (i) validates the moderate-deviation result of Theorem 2, (ii) illustrates the pooling efficiency gain predicted by the conditional variance formula (18), and (iii) assesses the quality of the normal approximation. All computations use $N=4\times 10^{4}$ replications (except the Q-Q study, which uses $2\times 10^{4}$), with a fixed random seed for reproducibility.

### 4.1. Simulation Design

We take the offspring law $X_{n,i}^{\left(j\right)}=1+\mathrm{Poisson}\left(\Lambda_{n}\right)$, where the environmental variable $\Lambda_{n}∼\mathrm{Gamma}\left(\mathrm{shape}=1,\mathrm{scale}=0.3\right)$ is drawn once per generation and shared by all *L* populations. This choice satisfies (H1) (each individual has at least one offspring) and yields the closed-form parameters

(23) $$m=1+E\left[\Lambda_{0}\right]=1.3,\;\sigma^{2}=\mathrm{Var}\left(\Lambda_{0}\right)=0.09,\;\tau^{2}=E\left[\Lambda_{0}\right]=0.30.$$

Since the sum of $Z_{n}^{\left(j\right)}$ conditionally independent $\mathrm{Poisson}(\Lambda_{n})$ variables is $\mathrm{Poisson}(Z_{n}^{\left(j\right)}\Lambda_{n})$, the recursion $Z_{n+1}^{\left(j\right)}=Z_{n}^{\left(j\right)}+\mathrm{Poisson}\left(Z_{n}^{\left(j\right)}\Lambda_{n}\right)$ is simulated exactly; for very large populations, the ratio $Z_{n+1}^{\left(j\right)}/Z_{n}^{\left(j\right)}$ concentrates at $1+\Lambda_{n}$ and is evaluated accordingly.

We deliberately work in the *weakly supercritical* regime m=1.3. As shown by (18), pooling reduces only the demographic term $\tau^{2}L^{-2}\sum_{j}1/Z_{k}^{\left(j\right)}$; for strongly supercritical processes, $Z_{k}$ grows so fast that $\sum_{k}E\left(1/Z_{k}\right)$ is a small constant and the gain is negligible. The weakly supercritical regime keeps $Z_{k}$ moderate over the observation window, making the 1/L efficiency gain clearly visible. Throughout, we set $n_{0}=0$.

### 4.2. Validation of the Moderate Deviation Result

**Figure 1:**
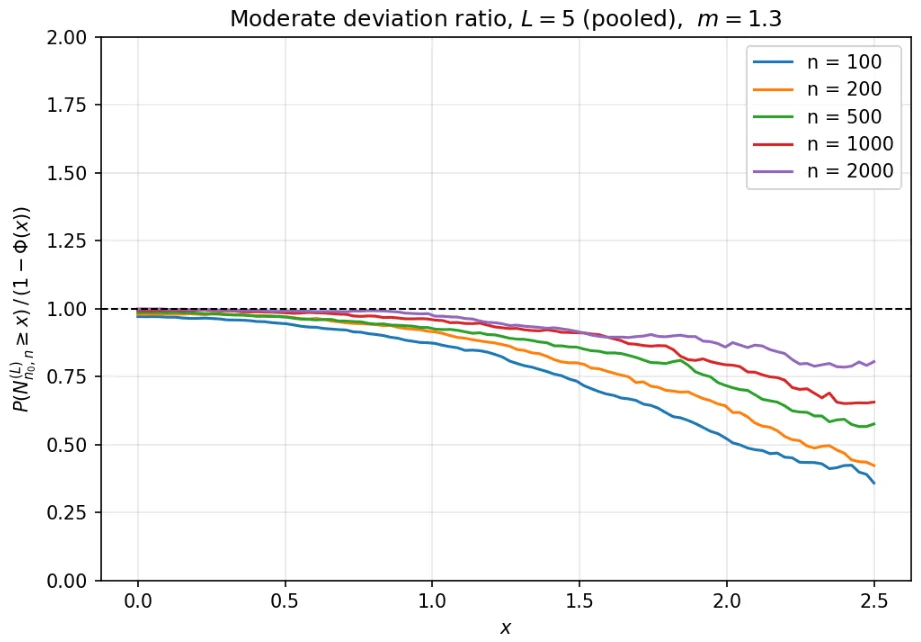
Tail-probability ratio $R\left(x\right)=P\left(N_{n_{0},n}^{\left(L\right)}\geq x\right)/\left(1-\Phi \left(x\right)\right)$ for the pooled *t*-statistic, L=5, m=1.3. The horizontal axis represents the deviation *x*, and the vertical axis gives the ratio $R\left(x\right)=P\left(N_{n_{0},n}^{\left(L\right)}\geq x\right)\;/\;\left(1-\Phi \left(x\right)\right)$. As *n* increases, the curves approach the horizontal line R≡1, illustrating Theorem 2.

Fixing L=5, we estimate the tail-probability ratio $R\left(x\right)=P\left(N_{n_{0},n}^{\left(L\right)}\geq x\right)/\left(1-\Phi \left(x\right)\right)$ for x∈[0,2.5] and n∈{100,200,500,1000,2000}; see Figure 1. Consistently with Theorem 2, R(x)→1 as *n* grows, uniformly over an expanding range of *x*. Table 1 reports R(x) at three representative points: the ratio increases monotonically toward 1 with *n*. For n=1000, the ratio exceeds 0.95 at x=1.0 and 0.79 at x=2.0, indicating that the pooled *t*-statistic already delivers reliable tail probability estimates at moderate sample sizes. This is notable given the weakly supercritical regime, under which single-population estimators typically require substantially larger *n* to achieve comparable accuracy.

### 4.3. Efficiency Gain as *L* Increases

**Figure 2:**
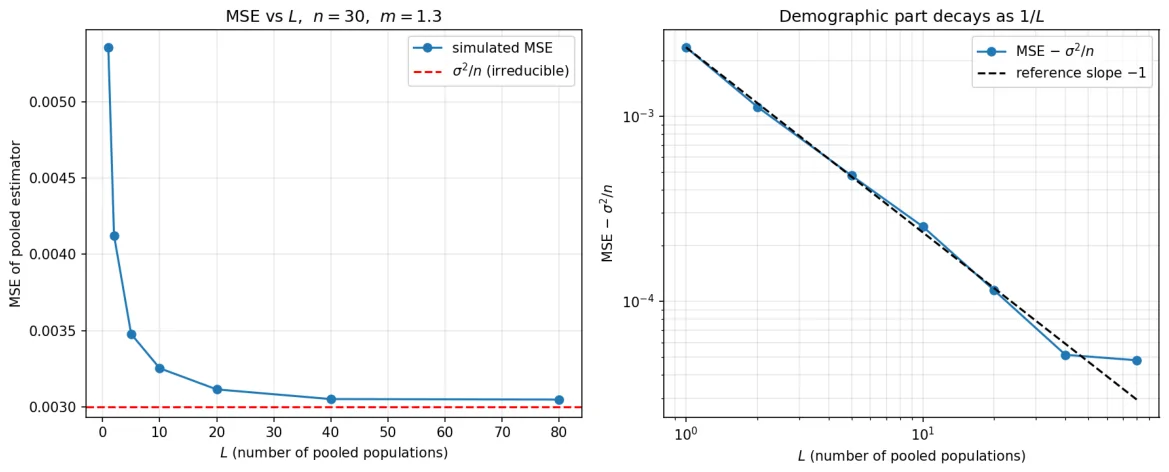
Left: MSE of the pooled estimator versus *L* (n=30, m=1.3), approaching the irreducible plateau $\sigma^{2}/n$ (dashed). Right: the excess $\mathrm{MSE}-\sigma^{2}/n$ on a log–log scale, tracking the reference slope −1, confirming the 1/L decay of the demographic variance.

Fixing n=30, we compute the mean squared error $\mathrm{MSE}\left(L\right)=E[{\left({\hat{m}}_{n_{0},n}^{\left(L\right)}-m\right)}^{2}]$ for L∈{1,2,5,10,20,40,80}; see Figure 2. Taking expectations in (18) and using the orthogonality of the martingale differences ${\tilde{\eta }}_{k+1}^{\left(L\right)}$ together with the identical distribution of the *L* populations (so that $\sum_{j=1}^{L}E\left(1/Z_{k}^{\left(j\right)}\right)=L\;E\left(1/Z_{k}\right)$), the variance of the estimator decomposes as

(24) $$\mathrm{Var}\left({\hat{m}}_{n_{0},n}^{\left(L\right)}\right)=\underset{\mathrm{environmental},\;\mathrm{irreducible}}{\underset{︸}{\frac{\sigma^{2}}{n}}}+\underset{\mathrm{demographic},\;\propto \;1/L}{\underset{︸}{\frac{\tau^{2}}{L\;n^{2}}\sum_{k=n_{0}}^{n_{0}+n-1}E\;\left(\frac{1}{Z_{k}}\right)}}.$$

The demographic term carries a factor 1/L rather than the $1/L^{2}$ of (18), precisely because the sum over the *L* populations contributes a factor *L*. Consequently, as *L* increases, the MSE decreases toward the irreducible plateau $\sigma^{2}/n=0.003$, while the excess $\mathrm{MSE}\left(L\right)-\sigma^{2}/n$ decays like 1/L. Table 2 confirms this: the product $L\cdot (\mathrm{MSE}\left(L\right)-\sigma^{2}/n)$ is nearly constant (≈$2.3\times 10^{-3}$) for L=1,…,40; the deviation at L=80 reflects the Monte Carlo resolution floor, the excess having fallen to $\approx 5\times 10^{-5}$. This confirms that even with a modest sample size of n=30, pooling L=40 populations already eliminates nearly all of the demographic component of the variance, leaving only the irreducible environmental floor $\sigma^{2}/n$. In practical terms, the pooled estimator approaches the theoretical optimal precision with only a moderate number of parallel trajectories. Moreover, the bias-variance decomposition in Table 2 shows that the empirical bias $E[{\hat{m}}^{\left(L\right)}]-m$ is of order $10^{-4}$ throughout, its largest magnitude being $4.1\times 10^{-4}$ at L=1, two orders of magnitude below the standard deviation. The estimator is therefore essentially unbiased, and the decay of the MSE is driven entirely by the reduction of the variance (indeed, $\mathrm{Var}({\hat{m}}^{\left(L\right)})$ and MSE(L) agree to the precision shown).

### 4.4. Robustness

To confirm that the pooling gain is not an artifact of a particular model choice, we repeat the efficiency experiment under three further scenarios, summarized in Table 3. Scenario S1 is the Gamma–Poisson baseline of Section 4.1. Scenario S2 changes the offspring mean to m=1.5 (and hence, the environmental variance to $\sigma^{2}=0.25$) by taking Λ∼Gamma(1,0.5). Scenario S3 replaces the Gamma environment by a log-normal one with parameters chosen so that *m*, $\sigma^{2}$, and $\tau^{2}$ remain exactly as in S1, thereby isolating the effect of the environmental distribution. Scenario S4 replaces the Poisson offspring law by a geometric one, X=1+Geometric(1/(1+Λ)), which leaves *m* and $\sigma^{2}$ unchanged but raises the demographic variance to $\tau^{2}=0.48$.

**Figure 3:**
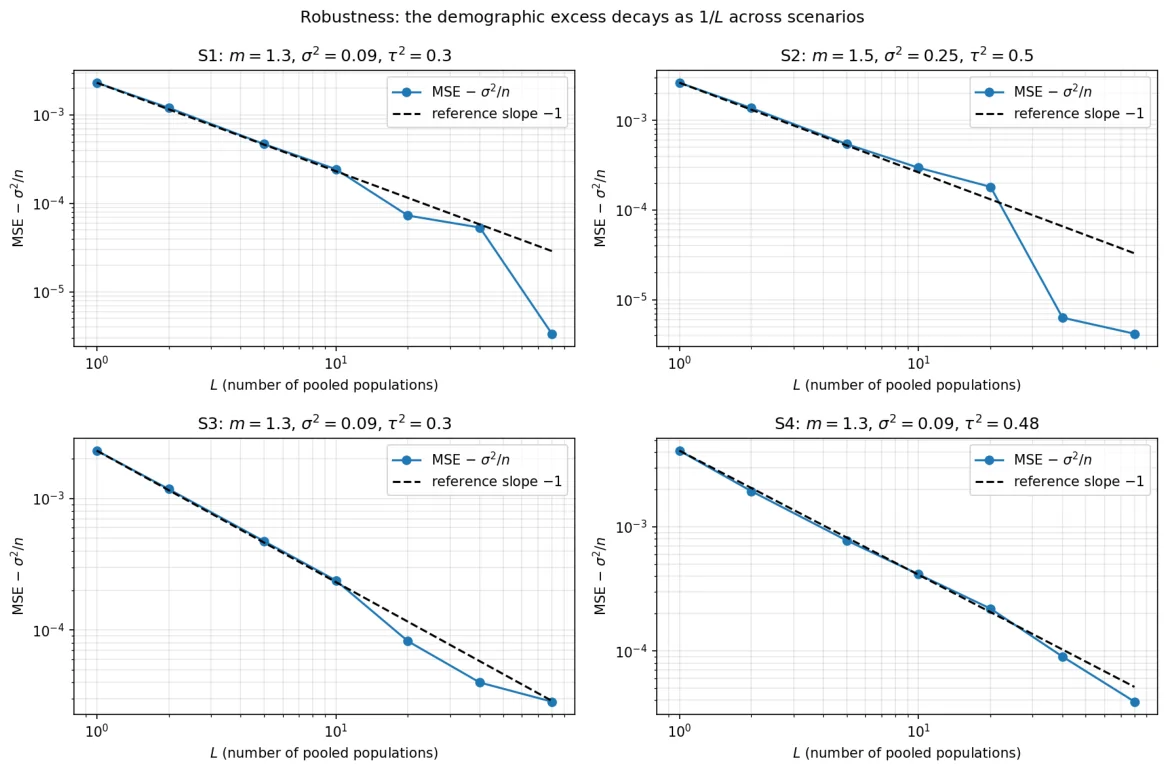
Robustness of the 1/L decay. Each panel shows the excess $\mathrm{MSE}\left(L\right)-\sigma^{2}/n$ against *L* on a log–log scale for one scenario, together with a reference slope of −1. The excess follows the −1 slope in all four scenarios, confirming that the demographic variance decays as 1/L regardless of the environmental distribution, offspring law, or parameter values.

In all four scenarios, the rescaled excess $L\cdot (\mathrm{MSE}\left(L\right)-\sigma^{2}/n)$ is approximately constant across *L* (Table 3 and Figure 3), confirming that the demographic component decays as 1/L. The magnitude of this constant is governed by $\tau^{2}$: scenarios S1 and S3, with $\tau^{2}=0.30$, yield essentially the same value, and S4, with $\tau^{2}=0.48$, yields the largest value. (In S2, the larger *m* accelerates the growth of $Z_{k}$ and, hence, the decay of $1/Z_{k}$, which partially offsets its larger $\tau^{2}$.) The robustness of the 1/L decay across environmental distributions, offspring laws, and parameter values supports the generality of the pooling efficiency gain.

### 4.5. Coverage of Confidence Intervals

Since confidence intervals for *m* are a key deliverable of Corollary 3, we verify their finite-sample performance. For several pairs (n,L), we construct the level-95% interval of Corollary 3 on each Monte Carlo replication and record the empirical coverage probability and the average interval length (Table 4). The coverage is close to the nominal level 95% and improves with *n* (from 93.1% at n=50 to 94.2% at n=200 for L=5). The slight shortfall is attributable to the finite-sample deviation from normality visible in the Q-Q plots. More importantly, for fixed *n*, the average interval length decreases as *L* increases (from 0.129 at L=1 to 0.117 at L=20 for n=100), which is the direct practical benefit of pooling: more populations yield shorter confidence intervals at essentially no cost in coverage. To make the interval concrete, at (n,L)=(200,5), the pooled estimator yields the level-95% interval 1.30±0.042, i.e., [1.26,1.34], which covers the true m=1.3.

### 4.6. Q-Q Plots

**Figure 4:**
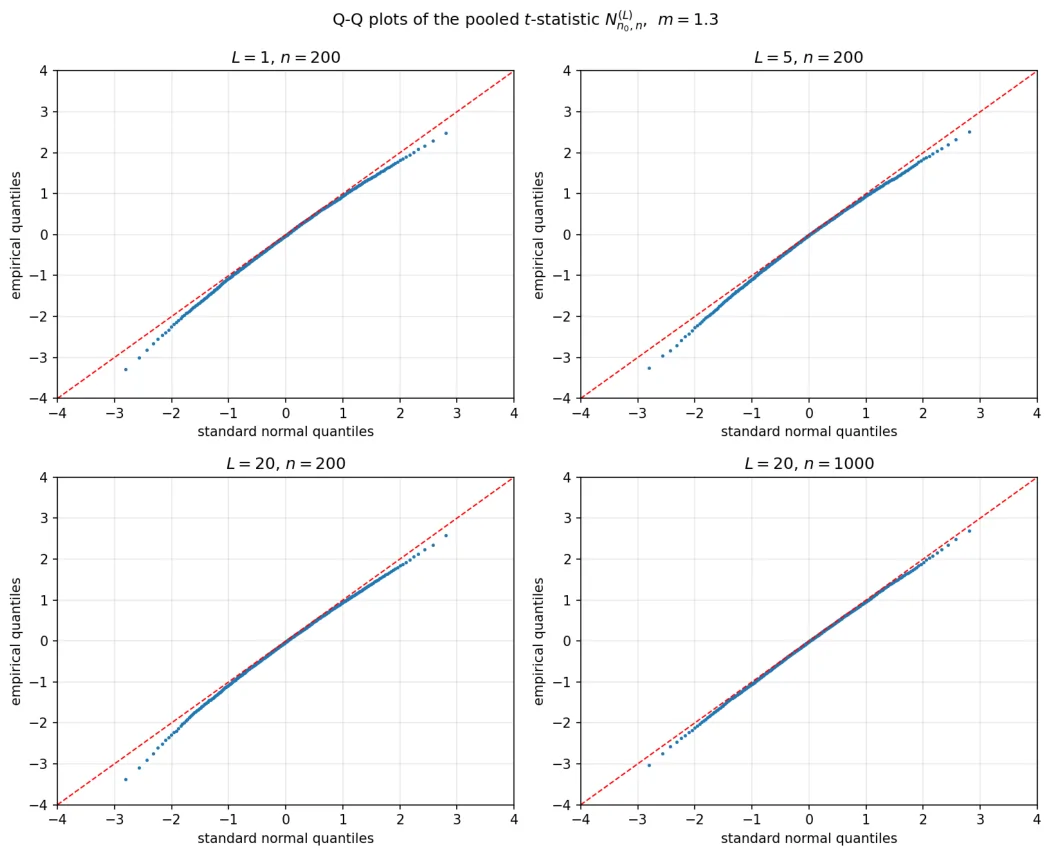
Normal Q-Q plots of the pooled *t*-statistic $N_{n_{0},n}^{\left(L\right)}$ for selected (L,n), m=1.3. Points near the diagonal indicate closeness to standard normality. The dashed red line is the reference diagonal y=x.

Figure 4 shows normal Q-Q plots of $N_{n_{0},n}^{\left(L\right)}$ for (L,n)∈{(1,200),(5,200),(20,200),(20,1000)}. The empirical quantiles align closely with the diagonal; the sample skewness and excess kurtosis, reported in Table 5, are small and decrease as *n* grows (from skewness −0.30, excess kurtosis 0.21 at n=200 to −0.12, 0.08 at n=1000), confirming that the normal approximation improves with the sample size, in agreement with Corollary 1.

### 4.7. Summary of the Numerical Findings

The simulation results confirm all qualitative predictions of the theory: (i) the moderate-deviation ratio R(x) tends to 1 (Theorem 2); (ii) pooling reduces the demographic component of the variance at the rate 1/L while leaving the environmental component $\sigma^{2}/n$ untouched, exactly as dictated by the conditional variance formula (18); and (iii) the normal approximation sharpens as *n* increases (Corollary 1).

## 5. Proofs

Throughout this section, we work under (H1)–(H5) and use the notation of Section 3. Recall $A_{k}^{\left(j\right)}=Z_{k+1}^{\left(j\right)}/Z_{k}^{\left(j\right)}-m$ and ${\tilde{\eta }}_{k+1}^{\left(L\right)}=L^{-1}\sum_{j=1}^{L}A_{k}^{\left(j\right)}$.

### 5.1. Proof of Theorem 1

We first record the single-population conditional moments. Fix *j* and condition on $\left(\xi_{k},F_{k}\right)$. Since $Z_{k+1}^{\left(j\right)}=\sum_{i=1}^{Z_{k}^{\left(j\right)}}X_{k,i}^{\left(j\right)}$, where $Z_{k}^{\left(j\right)}$ is $F_{k}$-measurable and the $X_{k,i}^{\left(j\right)}$ are i.i.d. with mean $m(\xi_{k})$ and variance $\mathrm{Var}(X_{k,1}^{\left(j\right)}|\xi_{k})$ given $\xi_{k}$,

(25) $$E\left[A_{k}^{\left(j\right)}|\xi_{k},F_{k}\right]=m(\xi_{k})-m,$$

(26) $$\mathrm{Var}\left(A_{k}^{\left(j\right)}|\xi_{k},F_{k}\right)\;=\frac{1}{{\left(Z_{k}^{\left(j\right)}\right)}^{2}}\;Z_{k}^{\left(j\right)}\;\mathrm{Var}\left(X_{k,1}^{\left(j\right)}|\xi_{k}\right)=\frac{1}{Z_{k}^{\left(j\right)}}\;\mathrm{Var}\left(X_{k,1}^{\left(j\right)}|\xi_{k}\right).$$

Hence,

$$E\left[{\left(A_{k}^{\left(j\right)}\right)}^{2}|\xi_{k},F_{k}\right]=\frac{1}{Z_{k}^{\left(j\right)}}\;\mathrm{Var}\left(X_{k,1}^{\left(j\right)}|\xi_{k}\right)+{\left(m\left(\xi_{k}\right)-m\right)}^{2}.$$

Taking expectation over $\xi_{k}$, which is independent of $F_{k}$ while $Z_{k}^{\left(j\right)}$ is $F_{k}$-measurable, and using $Z_{1}=X_{0,1}$ (so that $\tau^{2}=E\left[{\left(Z_{1}-m\left(\xi_{0}\right)\right)}^{2}\right]=E\left[\mathrm{Var}\left(X_{0,1}|\xi_{0}\right)\right]$) together with $\sigma^{2}=E\left[{\left(m\left(\xi_{0}\right)-m\right)}^{2}\right]$, we obtain the diagonal identity

(27) $$E\left[{\left(A_{k}^{\left(j\right)}\right)}^{2}|F_{k}\right]=\frac{\tau^{2}}{Z_{k}^{\left(j\right)}}+\sigma^{2},\;1\leq j\leq L.$$

For the cross-terms, fix i≠j and condition on $\left(\xi_{k},F_{k}\right)$. By the shared-environment and conditional-independence structure of (13), $Z_{k+1}^{\left(i\right)}$ and $Z_{k+1}^{\left(j\right)}$ are independent given $\left(\xi_{k},F_{k}\right)$, so by (25),

$$E\left[A_{k}^{\left(i\right)}A_{k}^{\left(j\right)}|\xi_{k},F_{k}\right]=E\left[A_{k}^{\left(i\right)}|\xi_{k},F_{k}\right]\;E\left[A_{k}^{\left(j\right)}|\xi_{k},F_{k}\right]={\left(m\left(\xi_{k}\right)-m\right)}^{2}.$$

Taking expectation over $\xi_{k}$ gives the cross identity

(28) $$E\left[A_{k}^{\left(i\right)}A_{k}^{\left(j\right)}|F_{k}\right]=\sigma^{2},\;i\neq j.$$

Note that (28) is independent of $Z_{k}^{\left(i\right)},Z_{k}^{\left(j\right)}$: the only coupling between distinct populations is through the common environment, which contributes exactly the environmental variance $\sigma^{2}$.

Finally, expanding ${\tilde{\eta }}_{k+1}^{\left(L\right)}=L^{-1}\sum_{j}A_{k}^{\left(j\right)}$ and combining (27) and (28),

$$E\left[{\left({\tilde{\eta }}_{k+1}^{\left(L\right)}\right)}^{2}|F_{k}\right]=\frac{1}{L^{2}}\left[\sum_{j=1}^{L}(\frac{\tau^{2}}{Z_{k}^{\left(j\right)}}+\sigma^{2})+\sum_{i\neq j}\sigma^{2}\right]=\frac{1}{L^{2}}[\tau^{2}\sum_{j=1}^{L}\frac{1}{Z_{k}^{\left(j\right)}}+L\sigma^{2}+L\left(L-1\right)\sigma^{2}],$$

and since $L\sigma^{2}+L\left(L-1\right)\sigma^{2}=L^{2}\sigma^{2}$, this equals $\sigma^{2}+\left(\tau^{2}/L^{2}\right)\sum_{j}1/Z_{k}^{\left(j\right)}$, proving (18). □

### 5.2. Proof of Lemma 1

By [19] (Equations (91) and (92); see also [24]), the single-population martingale differences obey the moment bound

(29) $$E\left[|A_{k}^{\left(j\right)}{|}^{2+\rho }|F_{k}\right]\leq C\;E\left[{\left(A_{k}^{\left(j\right)}\right)}^{2}|F_{k}\right],\;C=2^{1+\rho }\;\frac{E|Z_{1}-m\left(\xi_{0}\right){|}^{2+\rho }+E{\left|m\left(\xi_{0}\right)-m\right|}^{2+\rho }}{\sigma^{2}},$$

which is finite by (H4) and holds for every *j* (the populations being identically distributed). Since $x\mapsto {\left|x\right|}^{2+\rho }$ is convex and 2+ρ≥1, Jensen’s inequality gives $|{\tilde{\eta }}_{k+1}^{\left(L\right)}{|}^{2+\rho }=|L^{-1}\sum_{j}A_{k}^{\left(j\right)}{|}^{2+\rho }\leq L^{-1}\sum_{j}{\left|A_{k}^{\left(j\right)}\right|}^{2+\rho }$, whence, writing $v_{j}:=\tau^{2}/Z_{k}^{\left(j\right)}+\sigma^{2}$,

$$E\left[|{\tilde{\eta }}_{k+1}^{\left(L\right)}{|}^{2+\rho }|F_{k}\right]\leq \frac{1}{L}\sum_{j=1}^{L}E\left[|A_{k}^{\left(j\right)}{|}^{2+\rho }|F_{k}\right]\leq \frac{C}{L}\sum_{j=1}^{L}v_{j}=C(\sigma^{2}+\frac{\tau^{2}}{L}\sum_{j=1}^{L}\frac{1}{Z_{k}^{\left(j\right)}}).$$

Comparing with (18), and using that for $S:=\sum_{j}1/Z_{k}^{\left(j\right)}\geq 0$, the ratio $\left(\sigma^{2}+\left(\tau^{2}/L\right)S\right)/\left(\sigma^{2}+\left(\tau^{2}/L^{2}\right)S\right)$ lies in [1,L], we obtain

$$E\left[|{\tilde{\eta }}_{k+1}^{\left(L\right)}{|}^{2+\rho }|F_{k}\right]\leq CL\;E\left[{\left({\tilde{\eta }}_{k+1}^{\left(L\right)}\right)}^{2}|F_{k}\right]=:c_{1}\;E\left[{\left({\tilde{\eta }}_{k+1}^{\left(L\right)}\right)}^{2}|F_{k}\right],$$

with $c_{1}=CL$ finite since *L* is fixed. Setting $X_{i}={\tilde{\eta }}_{n_{0}+i}^{\left(L\right)}$ and $B_{n}^{2}=n\sigma^{2}$, the bound reads $E[|X_{i}{|}^{2+\rho }|F_{i-1}]\leq {\left(\gamma_{n}B_{n}\right)}^{\rho }\;E\left[X_{i}^{2}|F_{i-1}\right]$ with $\gamma_{n}=c_{1}^{1/\rho }/B_{n}≍n^{-1/2}$, so (A2) holds. Note that this step uses only the convexity of $x\mapsto {\left|x\right|}^{2+\rho }$ and no independence between the populations, so the shared-environment coupling introduces no additional difficulty here. □

### 5.3. Proof of Lemma 2

By (18), the conditional variance ${\langle S\rangle }_{n}:=\sum_{k=n_{0}}^{n_{0}+n-1}E\left[{\left({\tilde{\eta }}_{k+1}^{\left(L\right)}\right)}^{2}|F_{k}\right]$ satisfies, with $B_{n}^{2}=n\sigma^{2}$,

$${\langle S\rangle }_{n}-B_{n}^{2}=\frac{\tau^{2}}{L^{2}}\sum_{j=1}^{L}\sum_{k=n_{0}}^{n_{0}+n-1}\frac{1}{Z_{k}^{\left(j\right)}}\;\geq 0.$$

Fix x>0. Then, writing $c^{\prime }=L\sigma^{2}/\tau^{2}$,

$$P\left({|\langle S\rangle }_{n}-B_{n}^{2}|\geq xB_{n}^{2}\right)=P\;\left(\frac{1}{L}\sum_{j=1}^{L}\left(\frac{1}{n}\sum_{k=n_{0}}^{n_{0}+n-1}\frac{1}{Z_{k}^{\left(j\right)}}\right)\geq c^{\prime }x\right)\leq \sum_{j=1}^{L}P\;\left(\frac{1}{n}\sum_{k=n_{0}}^{n_{0}+n-1}\frac{1}{Z_{k}^{\left(j\right)}}\geq c^{\prime }x\right),$$

the last step being a union bound, which is valid under arbitrary dependence, so that the shared-environment coupling presents no additional difficulty at this step. Each summand is a single-population tail; by the bound established in the proof of [19] (Equation (89)) (see Lemma A2), there are constants $c_{\rho }>0$ such that for all y>0,

$$P\;\left(\frac{1}{n}\sum_{k=n_{0}}^{n_{0}+n-1}\frac{1}{Z_{k}^{\left(j\right)}}\geq y\right)\leq c_{\rho }^{-1}e^{10c_{\rho }/\mu }\exp\;\left(-c_{\rho }\;\frac{n}{\ln n}\;y\right).$$

Combining,

$$P\left({|\langle S\rangle }_{n}-B_{n}^{2}|\geq xB_{n}^{2}\right)\leq L\;c_{\rho }^{-1}e^{10c_{\rho }/\mu }\exp\;\left(-c_{\rho }c^{\prime }\;\frac{n}{\ln n}\;x\right)=c\;\exp\;\left(-x\;\delta_{n}^{-2}\right),$$

with $c=Lc_{\rho }^{-1}e^{10c_{\rho }/\mu }$ and

$$\delta_{n}^{2}=\frac{\tau^{2}}{c_{\rho }L\sigma^{2}}\cdot \frac{\ln n}{n}≍\frac{\ln n}{n}.$$

For *n* large, $\delta_{n}\in \left(0,1/4\right]$, so (A1) holds. The constant is proportional to 1/L, so that pooling sharpens the concentration as *L* increases. □

### 5.4. Proof of Theorem 2

Set $X_{i}={\tilde{\eta }}_{n_{0}+i}^{\left(L\right)}$ for i=1,…,n, so that $\left(X_{i},F_{n_{0}+i}\right)$ is a finite martingale difference sequence (Proposition 1) with ${\hat{m}}_{n_{0},n}^{\left(L\right)}-m=n^{-1}\sum_{i=1}^{n}X_{i}$, and let

$$W_{n}^{\left(L\right)}=\frac{\sum_{i=1}^{n}X_{i}}{{\left(\sum_{i=1}^{n}X_{i}^{2}\right)}^{1/2}}$$

be the associated self-normalized sum.

*Step 1 (reduction to $W_{n}^{\left(L\right)}$).* The pooled *t*-statistic (20) and $W_{n}^{\left(L\right)}$ are linked by the exact algebraic identity of Chung [23]: for all x≥0,

$$P\left(N_{n_{0},n}^{\left(L\right)}\geq x\right)=P\;\left(W_{n}^{\left(L\right)}\geq x{\left(\frac{n}{n+x^{2}-1}\right)}^{1/2}\right),$$

which holds because $N_{n_{0},n}^{\left(L\right)}$ is built from the martingale differences $X_{i}$ exactly as the classical *t*-statistic is built from its summands (cf. [19], Equation (18)).

*Step 2 (moderate deviations for $W_{n}^{\left(L\right)}$).* By Lemmas 1 and 2, $\left(X_{i},F_{n_{0}+i}\right)$ satisfies conditions (A1) and (A2) with $\gamma_{n}≍n^{-1/2}$ and $\delta_{n}^{2}≍\ln n/n$, exactly the parameter orders obtained for the single-population martingale in [19] (Section 6). We emphasize that the cross-population dependence induced by the shared environment enters the analysis only through the conditional variance, which is computed exactly in Theorem 1: the non-vanishing cross-terms give rise to the undiluted environmental term $\sigma^{2}$ in (18). The subsequent verification of (A2) and (A1) relies only on Jensen’s inequality and on a union bound, respectively, neither of which requires any independence between populations. Consequently, once (A1) and (A2) are established, the moderate-deviation theorem of [19] (Theorem 2.1), which requires nothing beyond these two conditions, applies to $W_{n}^{\left(L\right)}$ without modification and, through Step 1, yields (21) for all $0\leq x=o(\sqrt{n/\ln n})$.

*Step 3 (range of validity).* By Theorem 2.1 of [19], the relative error is 1+o(1) for $0\leq x=o\left(\min\{\gamma_{n}^{-\rho /(2+\rho )},\delta_{n}^{-1/2}\}\right)$. With $\gamma_{n}≍n^{-1/2}$, one has $\gamma_{n}^{-\rho /(2+\rho )}≍n^{\rho /(4+2\rho )}$, and with $\delta_{n}^{2}≍\ln n/n$, one has $\delta_{n}^{-1/2}≍{\left(n/\ln n\right)}^{1/4}$, which dominates $n^{\rho /(4+2\rho )}$ for ρ≤1. Hence, the binding constraint is $x=o\left(n^{\rho /(4+2\rho )}\right)$, giving (22).

*Step 4 (lower tail).* Since $\left(-X_{i},F_{n_{0}+i}\right)$ is also a martingale difference sequence satisfying the same conditions, Steps 1–3 apply with $-X_{i}$, giving the lower-tail statement. □

## 6. Discussion

### 6.1. Summary

We have extended the self-normalized Cramér moderate deviation theory of [19] from a single BPRE to *L* conditionally independent populations sharing a common environment. The pooled Lotka–Nagaev estimator and its martingale difference sequence lead to the conditional variance formula $\sigma^{2}+\left(\tau^{2}/L^{2}\right)\sum_{j}1/Z_{k}^{\left(j\right)}$, in which the environmental variance $\sigma^{2}$ is undiluted while the demographic variance is attenuated at rate $1/L^{2}$. Verifying the two conditions of the underlying martingale theorem with the same parameter orders as in the single-population case, we obtained self-normalized Cramér moderate deviations for the pooled *t*-statistic over the range $o(n^{\rho /(4+2\rho )})$, together with a Berry–Esseen bound, a moderate deviation principle, and confidence intervals for the offspring mean. The distinctive feature of the multi-population setting, a pooling efficiency gain in which the demographic variance is reduced like 1/L while the environmental variance is not, was confirmed numerically.

### 6.2. Extensions and Open Questions

1. **Weighted pooling.** If population sizes differ substantially, a weighted estimator ${\hat{m}}^{\left(w\right)}=\sum_{j}\sum_{k}w_{k}^{\left(j\right)}Z_{k+1}^{\left(j\right)}/Z_{k}^{\left(j\right)}$ with $w_{k}^{\left(j\right)}\propto Z_{k}^{\left(j\right)}$ may be more efficient. However, the cross-terms would then no longer reduce to the constant $\sigma^{2}$, so the clean structure of (18) is lost and a new analysis is required.
2. **The regime $L=L_{n}\to \infty$ (beyond (H5)).** If the number of populations grows with *n*, the union bound [25] in the proof of Lemma 2 becomes crude, and a sharper treatment of the joint tail of the $L_{n}$ conditionally independent populations (e.g., via extreme-value estimates) is needed. The interplay between the growth rate of $L_{n}$ and *n* may produce a phase transition in the moderate-deviation range; identifying the critical rate is an open problem.
3. **Two-sample problem.** When two groups of populations have possibly different offspring means $m\neq m^{\prime }$, the difference ${\hat{m}}^{\left(L\right)}-{\hat{m}}^{\prime (L^{\prime })}$ and its Studentized version invite a two-sample Cramér moderate-deviation result, of interest for testing the equality of offspring means across environments.
4. **Standardized (non-self-normalized) case.** Under stronger Bernstein-type conditions (cf. [19], Theorem 3.4), the standardized counterpart $S_{n_{0},n}^{\left(L\right)}=\sqrt{n}\;\left({\hat{m}}_{n_{0},n}^{\left(L\right)}-m\right)/\sigma$ should satisfy Cramér moderate deviations over the range $o(n^{1/6})$ with Berry–Esseen bound $O(\ln n/\sqrt{n})$.
5. **Pooling with immigration.** Combining the pooling of this paper with the immigration framework of [20], which refers to *L* independent Galton–Watson processes with immigration in a shared environment, would unify the two orthogonal extension directions. The conditional variance would then carry both the $\tau^{2}/L^{2}$ pooling term and an immigration-variance term ${\bar{v}}^{2}$.
6. **Partially correlated environments.** The irreducibility of the environmental variance relies on the idealized assumption that all *L* populations share exactly the same environment. If, more generally, the environmental means of two distinct populations are correlated with coefficient ϱ∈[0,1], the cross-terms become $\varrho \sigma^{2}$ rather than $\sigma^{2}$, and the pooled conditional variance takes the form $\sigma^{2}\left(1+(L-1)\varrho \right)/L+\left(\tau^{2}/L^{2}\right)\sum_{j}1/Z_{k}^{\left(j\right)}$. The extreme cases ϱ=1 and ϱ=0 recover, respectively, the undiluted environmental variance and the 1/L-diluted variance of independent environments. The pooling gain is thus governed by the environmental correlation ϱ, and extending the analysis to a general dependence structure is a natural direction for future work.
7. **Relaxing the no-extinction assumption.** The condition $p_{0}\left(\xi_{0}\right)=0$ a.s. in (H1) rules out extinction and confines the present results to the no-extinction idealization. Extending them to the usual supercritical regime $p_{0}>0$ requires conditioning on non-extinction, a standard but more delicate route that we leave to future work.

### 6.3. Practical Applicability

Beyond its theoretical interest, the pooled framework is directly motivated by settings in which several independent populations evolve under a common source of environmental randomness. Concrete examples include ecological monitoring of multiple sub-populations of a species in a shared habitat, replicated laboratory experiments such as parallel bacterial cultures in a common incubator, and epidemiological surveillance of communities subject to common climatic or policy shocks. In all these settings, the successive ratios $Z_{k+1}^{\left(j\right)}/Z_{k}^{\left(j\right)}$ are available from population counts, and the pooled estimator ${\hat{m}}^{\left(L\right)}$ provides a data-efficient way to estimate the common offspring mean *m*, which is the per-individual reproduction rate of the population.

Two caveats delimit the practical scope of the theory. First, assumption (H1) excludes extinction and is therefore appropriate only over observation windows short relative to the extinction time scale of the population. Second, the shared-environment structure idealizes reality, since the environments of distinct populations are typically highly correlated but not identical, while the partially correlated setting discussed above shows how the pooling gain degrades as the environmental correlation falls below one. Subject to these provisos, the confidence intervals of Corollary 3, together with the 1/L reduction of the demographic variance quantified in Section 4, provide a concrete and readily applicable recipe for pooling parallel population trajectories.

## Figures and Tables

**Table 1:** Tail-probability ratio R(x) at representative *x*, L=5, with Monte Carlo standard errors in parentheses (based on $4\times 10^{4}$ replications).

*n*	R(1.0)	R(1.645)	R(2.0)
100	0.873(0.011)	0.670(0.018)	0.518(0.024)
200	0.916(0.011)	0.755(0.019)	0.643(0.026)
500	0.928(0.011)	0.834(0.020)	0.716(0.028)
1000	0.959(0.011)	0.877(0.020)	0.790(0.029)
2000	0.974(0.011)	0.899(0.021)	0.864(0.031)

**Table 2:** Bias–variance decomposition of the MSE and the rescaled excess, n=30. The bias is two orders of magnitude below the standard deviation, so that $\mathrm{Var}\left({\hat{m}}^{\left(L\right)}\right)\approx \mathrm{MSE}\left(L\right)$; the near-constant last column evidences the 1/L decay of the variance-driven excess.

*L*	Bias	$\mathrm{Var}({\hat{m}}^{\left(L\right)})$	MSE(L)	$L\cdot (\mathrm{MSE}\left(L\right)-\sigma^{2}/n)$
	($\times 10^{-4}$)	($\times 10^{-3}$)	($\times 10^{-3}$)	($\times 10^{-3}$)
1	4.13	5.36	5.36	2.36
2	1.64	4.12	4.12	2.25
5	−2.57	3.48	3.48	2.39
10	0.18	3.25	3.25	2.53
20	−0.38	3.11	3.11	2.29
40	0.21	3.05	3.05	2.06
80	0.16	3.05	3.05	3.84

**Table 3:** Robustness of the 1/L decay across scenarios (n=30). The rescaled excess $L\cdot (\mathrm{MSE}\left(L\right)-\sigma^{2}/n)$, reported in units of $10^{-3}$ at L=1 and L=10, is nearly constant within each scenario, confirming the 1/L decay of the demographic variance.

Scenario	Model (Environment; Offspring)	*m*	$\sigma^{2}$	$\tau^{2}$	$L\cdot (\mathrm{MSE}-\sigma^{2}/n)$ ($\times 10^{-3}$, L=1/L=10)
S1	Gamma(1,0.3); 1+Poisson	1.3	0.09	0.30	2.32/2.44
S2	Gamma(1,0.5); 1+Poisson	1.5	0.25	0.50	2.63/2.96
S3	Log-normal; 1+Poisson	1.3	0.09	0.30	2.31/2.38
S4	Gamma(1,0.3); 1+Geometric	1.3	0.09	0.48	4.10/4.14

**Table 4:** Empirical coverage and average length of the level-95% confidence interval of Corollary 3 (based on $4\times 10^{4}$ replications).

(n,L)	Coverage (%)	Average Length
(100,1)	93.9	0.129
(100,5)	93.9	0.119
(100,20)	94.0	0.117
(50,5)	93.1	0.171
(200,5)	94.2	0.084

**Table 5:** Sample moments of $N_{n_{0},n}^{\left(L\right)}$.

(L,n)	Mean	Std. Dev.	Skewness	Excess Kurtosis
(1,200)	−0.065	1.020	−0.303	0.214
(5,200)	−0.068	1.025	−0.304	0.220
(20,200)	−0.069	1.026	−0.298	0.320
(20,1000)	−0.035	1.010	−0.122	0.077

## Data Availability

The simulation code used to generate all numerical results and figures in this study is available from the corresponding author upon reasonable request. The model parameters are fully reported in Section 4.1.

## Appendix A. Auxiliary Results from Fan and Shao (2025) [19]

For completeness, we record two single-population facts invoked in Section 5; both are established in [19] (Section 6) under (H1)–(H4) and, being properties of a single population, hold for each of our *L* identically distributed populations.

**Lemma A1** (Harmonic moment)**.** *There exists a constant α>0 such that $E[V^{-\alpha }]<\infty$, where $V=\lim_{n}V_{n}$ is the a.s. limit of the normalized process *(12)*.*

**Lemma A2** (Tail of the inverse-population sum)**.** *There is a constant $c_{\rho }>0$ such that for all x>0,*

$$P\;\left(\frac{1}{n}\sum_{k=n_{0}}^{n_{0}+n-1}\frac{1}{Z_{k}}\geq x\right)\leq c_{\rho }^{-1}\;e^{10c_{\rho }/\mu }\exp\;\left(-c_{\rho }\;\frac{n}{\ln n}\;x\right).$$

Lemma A2 is the single-population input to the union-bound argument in the proof of Lemma 2, and Lemma A1 underlies the moment bound (29) used in the proof of Lemma 1.
